# Global risks, the macroeconomy, and asset prices

**DOI:** 10.1007/s00181-022-02205-9

**Published:** 2022-02-14

**Authors:** Michele Costola, Michael Donadelli, Luca Gerotto, Ivan Gufler

**Affiliations:** 1 Department of Economics, Ca’ Foscari University of Venice, Venice, Italy; 2 Department of Economics and Management, University of Brescia, Brescia, Italy; 3 Department of Economics and Finance, Università Cattolica del Sacro Cuore, Largo Francesco Vito 1, 00168 Rome, Italy; 4 Department of Economics and Finance, LUISS Guido Carli, Rome, Italy

**Keywords:** Global risks, Uncertainty, Google searches, Macrodynamics, Asset prices, C32, D80, E32, G12

## Abstract

We propose a novel index of global risks awareness (GRAI) based on the most concerning risks—classified in five categories (economic, environmental, geopolitical, societal, and technological)—reported by the World Economic Forum (WEF) according to the potential impact and likelihood occurrence. The degree of public concern toward these risks is captured by Google search volumes on topics having the same or similar wording of that one of the WEF Global Risk Report. The dynamics of our GRAI exhibits several spillover episodes and indicates that concerns on the five different categories of global risks are—on average—highly interconnected. We further examine the interconnection between global risks perceptions and the macroeconomy and find that concerns on economic-, geopolitical-, and societal-related risks are net shock transmitters, whereas the macroeconomic variables are largely net receivers. Finally, we perform standard cross-sectional asset pricing tests and provide evidence that rising interconnection among global risks awareness commands a positive and statistically significant risk premium.

## Introduction

It is undeniable that the globalization process and the related increase in the degree of interdependence (or “connectedness") among countries have provided several benefits over the years. Globalization, however, may also lead to bad news. In particular, it has been found to amplify the international transmission of shocks and local risks. As a result, local risks/shocks have today a global dimension. But, even more importantly, the concern and awareness of local risks/shocks have a global dimension. This is also due to a wider and faster information flow. The ongoing pandemic has of course exacerbated the existing geopolitical, societal, and climate change challenges. This has called for further attention to the understanding of the major risks induced by mutating geopolitical, societal, and climate scenarios. Little attention, however, has been paid to how the most concerning global risks interact over time. Little is also known about the evolution of the concerns and awareness of major global risks.

In this paper, we aim to fill this gap by examining the degree of interdependence among the most concerning global risks identified by the World Economic Forum (WEF) for the period between 2007 and 2019. To the best of our knowledge, this is the first empirical work to focus on this set of risks. The WEF classifies these most concerning global risks in five different categories: (i) economic, (ii) environmental, (iii) geopolitical, (iv) societal, and (v) technological. Awareness or concern in these risks over time is captured by Google search volume indexes (SVI).^1^ The dynamics of the degree of connectedness among the different global risks is then computed using the standard methodology of Diebold and Yılmaz (2009, 2012). By doing so, we are able to build a novel index of interdependence among most concerning global risks. We refer to this index as the global risks awareness index (GRAI). We build our GRAI by relying first on a system in which only the dynamics of public attention to most concerning global risks (i.e., SVI) are included and then on a system in which main macroeconomic variables (i.e., unemployment, industrial production, inflation, business, and consumer confidence) are added to global risks perceptions. To gain more insights on the interaction among concerns to the different categories of risk as well on the interaction between global risks perceptions and macroeconomic fundamentals, the dynamics of net directional spillovers are also computed.

Our newly developed GRAI indicates that the population’s concerns about the different categories of risk are highly connected over time. In other words, a rising concern in one risk category tends to make people more concerned about other risks. Specifically, we find that, on average, 50% of the forecast error variance at the 6-month horizon originates from spillovers among the different risk categories. The inclusion of macroeconomic variables into the system does not alter our main findings, suggesting thus that concerns for different categories of risk and macroeconomic variables are—on average—highly connected.

The estimated net directional spillover indices suggest also that awareness of most concerning global risks is strongly transmitted to macroeconomic dynamics. In particular, concerns on economic-, political-, and societal-related risks are found to be the strongest net contributors to the system. On the contrary, we find macroeconomic variables to be—on average—net receivers. Surprisingly, the interaction among concerns of the different risk categories and the macroeconomy follows a declining path, suggesting that perceptions of the five risks are progressively deviating from the underlying macroeconomic fundamentals.

We finally test whether changes in the degree of connectedness among most concerning global risks are priced in the cross section of international returns. Results from standard cross-sectional asset pricing tests indicate that—on average—rising global risks awareness carries a positive risk premium.

The rest of the paper is organized as follows. Section 2 discusses the related literature. Section 3 describes the data used for the development of our dynamic measures of interconnectedness among the most concerning global risks. The methodology employed to build the global risks awareness index and obtain the directional contributions is then described in Sect. 4. Empirical findings from dynamic and static network analyses and cross-sectional asset pricing tests are then reported in Sect. 5. Section  6 concludes.

## Related literature

*Google search volumes and uncertainty* Our work is most closely connected to the growing literature that employs Google Trends data to study the relationship between investors/consumers sentiment and macroeconomic/financial dynamics. Bontempi et al. (2021), for instance, use the frequency of internet searches to build an index of economic policy uncertainty (EURQ). In line with the seminal work of Baker et al. (2016), they rely on internet search intensity for 183 policy-relevant terms. Bontempi et al. (2021) show that their Google Trends-based index captures people’s interest to acquire more information on one or more topics. Hence, EURQ can serve as a proxy for the level of perceived economic uncertainty.

Via standard VAR estimations, Donadelli (2015) and Castelnuovo and Tran (2017) analyze the effects of Google searches for macroeconomic policy-related topics on the US economy. They both find that rising concerns to macroeconomic policy-related issues generates significant adverse effects on real economic activity. Donadelli and Gerotto (2019) and Donadelli et al. (2020a) examine instead the macroeconomic and financial implications of rising population’s interest in non-economics-related topics. In a VAR framework, they find that an unexpected rise in the frequency of internet searches for non-economics-related topics can be detrimental for both production and employment. Bilgin et al. (2019) develop an index of economic and financial uncertainty for Turkey by employing Google search volumes for a set of 84 economics-related words. Their novel index of economic and financial uncertainty is found to be a good predictor of Turkish exchange rate, stock market, and interest rate dynamics, as well as the unemployment rate. Koop and Onorante (2019) employ instead Google Trends data to forecast macroeconomic variables using dynamic model selection (DMS) methods.

Indicators of rising population attention to specific topics—captured by Google search volumes—have also been used to examine the effects of changes in investor mood on financial market dynamics. In this respect, it has been largely observed that stock markets are sensitive to investors sentiment (see, among others, Marfatia 2020), which in turn is sensitive to online information availability (Xu et al. 2021). Dzielinski (2012) finds that weekly searches for the word “economy” predict transitory lower stock returns and higher realized volatility in the following week. Da et al. (2011) focus on searches for the name and the ticker symbol of companies that are part of Russell 3000, finding that increasing attention predicts a temporary rise in prices, followed by a price reversal. Da et al. (2015) focus on aggregate market returns and build a Financial and Economic Attitudes Revealed by Search index (FEARS), based on a set of keywords concerning economic conditions sentiment. The authors find that FEARS predicts low market returns today and high returns tomorrow. Bijl et al. (2016) employ S&P500 data and find that it would be profitable to sell highly searched stocks, and buy infrequently searched stocks. Recently, Kim et al. (2019) has shown that search volumes on specific companies, by means of Google Trends indexes, predict increased volatility. Similarly, Audrino et al. (2020) argue that attention to financial-market-related topics improves the goodness of realized volatility forecasts. Prange (2021) reports that search volumes concerning stock-, commodity-, and energy markets drive the time-varying correlation between asset returns. Gao et al. (2020) construct a weekly measure of sentiment for 38 countries and show that both global and country-level sentiment measures have a relevant role on stock market returns. Tosun (2021) finds that increasing investors’ attention after a cyberattack induces short-lived market reactions. Using Google searches for the topic “*coronavirus*”, Costola et al. (2021) observe that a rising interest in the pandemics helps to predict stock market returns. Lyocsa et al. (2020) retrieve search volume indices for 19 coronavirus pandemic-related English words. They then aggregate the individual search volume indicators to build a single indicator of coronavirus-related fear/uncertainty. This is found to have significant predictive power for future stock market uncertainty. John and Li (2021) construct sentiment indicators through search volumes for five categories of news (COVID, Market, Lockdown, Banking, and Government relief efforts) and analyze how the former influence price dynamics in stock and option markets.

*Media coverage & uncertainty.* Our work is then more distantly related to all those empirical works examining the relationship between real economic activity, financial dynamics, and risk/uncertainty. In the spirit of Baker et al. (2016), these studies rely on news-based indicators of macroeconomic policy uncertainty. The underlying idea is that increased media coverage on economic-policy-related issues alters the general public mood and thus their consumption and investment decisions. In particular, the bad mood implied by the rising frequency of economic-policy-related news has been found to undermine production, employment and equity valuations. A non-exhaustive list of works on the implications of rising news-based uncertainty for macroeconomic and financial dynamics includes the works of Tobback et al. (2018), Sahinoz and Erdogan Cosar (2018), Ghirelli et al. (2019), Donadelli et al. (2020b), Huang and Luk (2020), Lee et al. (2020), Aguilar et al. (2021), Ifwarsson et al. (2021) and Yu et al. (2021). In a similar fashion, Caldara and Iacoviello (2021) construct a novel measure of adverse geopolitical events based on the number of articles covering geopolitical tensions that appeared in the electronic archives of ten newspapers. In line with existing evidence on rising economic policy uncertainty, higher geopolitical risk is associated with adverse economic effects (i.e., a drop in investment and employment).

We differ from these empirical works in several aspects. First, we do not exclusively focus on a single category of risks (e.g., economic-policy-related risks or financial-related risks). Instead, we focus on five global risk categories, considering those global risks defined by the WEF as the most concerning ones, either by impact or by likelihood. Our novel index of risk awareness is thus meant to capture different categories of risks. Second, we are not interested in the effects of a specific news shock on macroquantities or financial markets. Differently, we construct an index representing the degree of interdependence among the five different categories of global risks. Additionally, we investigate the connectedness among global risks and major macroeconomic variables. Finally, as by the WEF classification, we do not focus exclusively on macroeconomic policy-related risks.

## Data

### Global risks

Since 2006, the World Economic Forum (WEF) drafts a yearly Global Risk Report (GRR). Since 2007, the GRR provides an analysis and ranking of different global risks. Via surveys, the different global risks are classified by impact and likelihood occurrence. The top five global risks by likelihood occurrence and impact reported in the different GRR editions are listed in Tables 1 and 2, respectively. Furthermore, global risks are classified into five different categories: economic, environmental, geopolitical, societal, and technological.

**Table 1:** Top five global risks (by Impact). *Source*: the Global Risk Report of the World Economic Forum, from the 2nd (2007) to the 14th (2019) editions

Edition	1st	2nd	3rd	4th	5th
2007	Asset price collapse	Retrenchment from globalization	Interstate and civil wars	Pandemics	Oil price shock
2008	Asset price collapse	Retrenchment from globalization (developed)	Slowing Chinese Economy	Oil and Gas price spike	Pandemics
2009	Asset price collapse	Retrenchment from globalization (developed)	Oil price shock	Chronic disease	Fiscal crises
2010	Asset price collapse	Retrenchment from globalization (developed)	Oil price shock	Chronic disease	Fiscal crises
2011	Fiscal crises	Climate change	Geopolitical conflict	Asset price collapse	Extreme energy price volatility
2012	Major systemic financial failure	Water supply crises	Food shortage crises	Chronic fiscal imbalances	Extreme volatility in energy and agricultural prices
2013	Major systemic financial failure	Water supply crises	Chronic fiscal imbalances	Diffusion of weapons of mass destruction	Failure of climate change adaptation
2014	Fiscal crises	Climate change	Water crises	Unemployment and underemployment	Critical information infrastructure breakdown
2015	Water crises	Rapid and massive spread of infectious diseases	Weapons of mass destruction	Interstate conflict with regional consequences	Failure of climate change mitigation and adaptation
2016	Failure of climate change mitigation and adaptation	Weapons of mass destruction	Water crises	Large scale involuntary migration	Severe energy price shock
2017	Weapons of mass destruction	Extreme weather events	Water crises	Major natural disasters	Failure of climate change mitigation and adaptation
2018	Weapons of mass destruction	Extreme weather events	Natural disasters	Failure of climate change mitigation and adaptation	Water crises
2019	Weapons of mass destruction	Failure of climate change mitigation and adaptation	Extreme weather events	Water crises	Natural disasters

**Table 2:** Top five global risks (by Likelihood). *Source*: the Global Risk Report of the World Economic Forum, from the 2nd (2007) to the 14th (2019) editions

Edition	1st	2nd	3rd	4th	5th
2007	Breakdown of critical information infrastructure	Chronic disease in developed countries	Oil price shock	China hard economic landing	Asset price collapse
2008	Asset price collapse	Middle East instability	Failed and falling states	Oil and Gas price spike	Chronic disease in developed countries
2009	Asset price collapse	Slowing Chinese Economy	Chronic disease	Global governance gaps	Retrenchment from globalization (emerging)
2010	Asset price collapse	Slowing Chinese Economy	Chronic disease	Fiscal crises	Global governance gaps
2011	Storms and cyclones	Flooding	Corruption	Biodiversity loss	Climate change
2012	Severe income disparity	Chronic fiscal imbalances	Rising greenhouse gas emissions	Cyberattacks	Water supply crises
2013	Severe income disparity	Chronic fiscal imbalances	Rising greenhouse gas emissions	Water supply crises	Mismanagement of population aging
2014	Income disparity	Extreme weather events	Unemployment and underemployment	Climate change	Cyberattacks
2015	Interstate conflict with regional consequences	Extreme weather events	Failure on national governance	State collapse or crises	High structural unemployment or underemployment
2016	Large-scale involuntary migration	Extreme weather events	Failure of climate change mitigation and adaptation	Interstate conflict with regional consequences	Major natural catastrophes
2017	Extreme weather events	Large scale involuntary migration	Major natural disasters	Large-scale terrorist attacks	Massive incident of data fraud/theft
2018	Extreme weather events	Natural disasters	Cyberattacks	Data fraud or theft	Failure of climate change mitigation and adaptation
2019	Extreme weather events	Failure of climate change mitigation and adaptation	Natural disasters	Data fraud or theft	Cyberattacks

Our aim is to capture the evolution of the world population’s attention to the most concerning global risks, as identified by the WEF in the GRR. To capture the world’s population’s attention to a specific risk, we use the frequency of internet searches (i.e., Google search volumes). Specifically, we make use of Google Trends “topics”, which allow to group searches for terms that share the same concept in any language (see also Kim et al. 2019). Thus, the use of “topics” rather than specific words (“search terms”, in the Google Trends glossary) allows overcoming the language issue.^2^ For each global risk—classified as most concerning by the WEF—we look for a match with a closely related topic among the ones available in Google Trends. To do so, two different criteria are employed. First, we select a topic having the exact wording of the global risk reported in GRR or one with similar wording (e.g., *cyberattacks*). In the absence of such a topic, our second criterion applies, that is, we identify concerns on one of the global risks identified by the WEF by means of “search terms”, and exploit the “related topics” function to make a shortlist of topics which are closely related to the chosen risk.^3^ We then select the topic with the highest average search volume within this shortlist.^4^ Note that, in some cases, the application of the second criterion forces us to choose two topics. This, for instance, when capturing concerns on risks related to energy price volatility, for which we consider both the topics “*petroleum*” and “*natural gas*”. Similarly, for the risk related to “*storms and cyclones*” and “*extreme weather events*”, we consider both the topics “*storm*” and “*cyclone*”. The structure of this matching process is shown in Table 3.^5^

**Table 3:** Google trends topics for the five global risk categories

Category	Google Trends “topic"	Google Trends “keyword"	Corresponding Risk(s) in the GRR
Technological	**Information Infrastructure**	/m/065ymc	**Breakdown of critical information infrastructure; critical information infrastructure breakdown**
	**Cyberattack**	/m/0p78w_d	**Cyberattacks**
	**Data theft**	/m/0814xz	**Data fraud or theft; massive incident of data fraud/theft**
Societal	Chronic condition	/m/0c1dj4	Chronic diseases in developed countries; chronic diseases
	Economic inequality	/m/020d7_	Severe economic disparity; income disparity
	**Migration**	/m/01gcn2	**Large involuntary migration**
	**Population ageing**	/m/035mf_	**Mismanagement of population aging**
	Water scarcity	/m/0dtw64	Water supply crises; water crises
	**Pandemic**	/m/061s4	**Pandemics**
	**Infectious disease**	/g/122891d2	**Rapid and massive spread of infectious diseases**
	Starvation	/m/01flyj	Food shortage crises
Economic	Petroleum; Natural gas	/m/05r_j; /m/05k4k	Oil price shock; Oil and gas price spike; severe energy price shock; extreme energy price volatility; extreme volatility in energy and agricultural prices
	**People’s Republic of China economy**	/m/011b4qhw	**China hard economic landing; slowing Chinese economy**
	Financial market	/m/0bjf2	Asset price collapse; major systemic financial failure
	**Globalization**	/m/0cjr0	**Retrenchment from globalization; Retrenchment from globalization (developed)**
	**Fiscal imbalance**	/m/05mrjy	Fiscal crises; **dynamic fiscal imbalances**
	**Unemployment**	/m/07s_c	**Unemployment and underemployment; High structural unemployment and underemployment**
Geopolitical	**Middle East**	/m/04wsz	**Middle East instability**
	**Failed state**	/m/012dw2	**Failed and falling states**; state collapse or crises
	**Global governance**	/m/067q10	**Global governance gaps**
	**Corruption**	/m/09pngm	**Corruption**
	Conflict	/m/0n5w902	Interstate and civil wars; Geopolitical conflict; interstate conflict with regional consequences
	Local government	/m/0dw5f	Failure on national governance
	Terrorism	/m/07jq_	Large-state terrorist attacks
	**Weapon of mass destruction**	/m/0dyq7	**Weapons of mass destruction; diffusion of weapons of mass destruction**
Environmental	Storm; Cyclone	/m/0z71l; /m/0brl6	Storms and cyclones; extreme weather events
	**Biodiversity**	/m/0c8g5	**Biodiversity loss**
	**Climate change**	/m/0cs9q	**Climate change; failure of climate change adaptation; failure of climate change adaptation and mitigation**
	**Greenhouse effect**	/m/0380q	**Rising greenhouse gas emissions**
	**Natural disasters**	/m/0g2k1	**Major natural disasters; natural disasters**

1st column: risk category; 2nd column: Google Trends “topic"; 3rd column: related “keywords"; 4th column: corresponding risk(s) in the GRR. **Bold** indicates the use of the first criterion (i.e., Google Trends topic having the same wording of the global risk reported in the GRR is available)

Thus, for each topic listed in Table 3, we retrieve from Google Trends the corresponding search volume index (SVI). Individual SVIs are then aggregated to build a unique SVI for each risk category (i.e., economic, environmental, geopolitical, societal, and technological) identified by the GRR. In practice, for each category of risk, we obtain (i) a SVI based on the global risks identified to have the strongest impact (“by impact”) and (ii) a SVI based on the global risks classified as those that are most likely to occur (“by likelihood”). As pointed out by the recent literature, each SVI can be interpreted as a measure of world population attention toward a specific category of risk. Importantly, the built SVI can also serve as a proxy for uncertainty related to the specific category of risk.

**Fig. 1:**
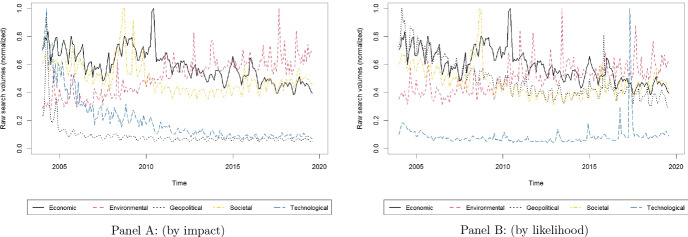
“Raw” SVIs. *Notes: *Panel A: Global risks by impact, Panel B: Global risks by likelihood. The figure reports the evolution of the World ”Raw" Google search volume index (SVI) for each risk category: (*i*) Economic, (*ii*) Environmental, (*iii*) Geopolitical, (*iv*) Societal and (*v*) Technological. Data are normalized such that the peak of each series corresponds to a value of one. All SVIs are monthly and run from January 2004 to August 2019

In order to aggregate different SVIs, the SVIs for the topics belonging to the same category have to be retrieved via a single query. By doing so the different SVIs can be consistently compared.^6^ The final SVI for each category of risk is computed through a vertical aggregation of the SVIs associated with the topics belonging to that category.^7^ All SVIs span the period January 2004–August 2019.^8^ Our “raw” aggregate SVIs for the different categories of risk are shown in Fig. 1. For the sake of robustness, following the approach of Da et al. (2011), we also compute normalized SVIs (“adj" SVIs). Descriptive statistics for both “raw" and “adj" SVIs are provided in Table 8 in Appendix.

### Macroeconomic variables

To capture the global economic conditions, we employ the following macro aggregates and prices for the OECD region: harmonized unemployment rate (*UR*), industrial production index (*IP*), and consumer price index (*CPI*). For the sake of completeness and in order to control for potential changes in aggregate consumers’ and firms’ sentiment, a consumer confidence indicator (*CCI*) and a business confidence indicator (*BCI*) are also considered. All employed series have been retrieved from the OECD database and run from January 2004 to August 2019.

## Methodology

In this section, we outline the popular approach developed by Diebold and Yılmaz (2009, 2012) to build a measure of total “connectedness” in a dynamic system of random variables (i.e., spillover index). The index is based on the forecast error variance decomposition (FEVD). FEVD shows the proportion of variations overtime in one variable induced by its own shocks, and that generated by shocks in the other variables in the VAR, by quantifying how much of the total variance forecast is attributed to each. A relatively high value of the spillover index implies that a larger proportion of the variables’ variations can be attributed to other variables’ shocks rather than to their own shocks. This approach allows us to measure how much of the future uncertainty concerning a given (global) risk category *i*, or macroeconomic variable *i*, depends on shocks coming from risk category attention/macroeconomic variable *j* at a given horizon *h*. The variance decomposition is based on the vector autoregressive process of order *p*,

1 $$\begin{aligned} {\mathbf {x}}_t = \sum _{l=1}^{p}\varvec{\Phi }_l {\mathbf {x}}_{t-l}+\varvec{\varepsilon }_t, \end{aligned}$$

where $\mathbf {x_t}=\left( x_{1,t},x_{2,t},\ldots ,x_{N,t},\right)$ is a random vector collecting the series of changes for each risk category attention/macroeconomic variable *i* for $i = 1, 2,\ldots ,N$, $\varvec{\Phi }_l$ is the $\left( N\times N\right)$ autoregressive matrix parameters at the *l*–th lag and $\varvec{\varepsilon }\sim {\mathsf {N}}\left( 0,\varvec{\Sigma }\right)$ is the vector of error terms that are assumed to follow a multivariate Gaussian distribution with variance–covariance matrix $\varvec{\Sigma }$. Given the covariance stationary property of the series of changes, we can represent the VAR(*p*) as an infinity moving average process

2 $$\begin{aligned} {\mathbf {x}}_t = \sum _{m=0}^{\infty }\varvec{\Gamma }_m\varvec{\varepsilon }_{t-m} \end{aligned}$$

where $\varvec{\Gamma }_m$ is a recursive matrix $\varvec{\Gamma }_m=\varvec{\Phi }_1\varvec{\Gamma }_{m-1}+\varvec{\Phi }_2\varvec{\Gamma }_{m-2}+\ldots + \varvec{\Phi }_p\varvec{\Gamma }_{m-p}$ for $m>0$, an $N\times N$ identity matrix for $m=0$, and equal to zero for $m<0$. Using the variance decomposition approach, the matrix $\varvec{\Gamma }_m$ of the moving average representation allows to analyze the variance of the forecast error of each variable by identifying all the parts which are originated from the shocks of all the other variables. As in Diebold and Yılmaz (2012), we use the generalized forecast error variance decomposition of Koop et al. (1996) and Pesaran and Shin (1998) which is invariant to the ordering of the variables in the VAR. The spillover represents the cross-variance share which is defined as the fraction of the H-step-ahead generalized error variance in forecasting $x_j$ coming from shocks to $x_i$ for $i,j = 1, 2, \ldots ,N$, such that $i\ne j$. The contribution of risk category attention/macroeconomic variable *i* to risk category attention/macroeconomic variable *j*’s H-step-ahead generalized forecast error variance is

3 $$\begin{aligned} \theta _{ij}(H) = \frac{\sigma _{jj}^{-1}\sum _{h=0}^{H-1}\left( e'_iA_h\varSigma e_j\right) ^2}{\sum _{h=0}^{H-1}\left( e'_iA_h\varSigma A'_h e_i\right) }, { \, } H = 1,2,\cdots , \end{aligned}$$

where $\varSigma$ is the covariance matrix of the vector of error terms $\varvec{\varepsilon }$, $\sigma _{jj}$ is the standard deviation of the error term $\varepsilon _j$ belonging to the *j*th equation in the system, and $e_i$ is the selection vector with the *i*th element equal to one and zeros otherwise. In this framework, the shocks to each variable are not orthogonalized and, thus, the contributions to the variance of the forecast error, in general, do not sum up to one. That is, $\sum _{j=1}^{N}\theta _{ij}(H)\ne 1$. Contributions $\theta _{ij}$ are therefore normalized by the row sum,

4 $$\begin{aligned} {\tilde{\theta }}_{ij}(H) = \frac{\theta _{ij}(H)}{\sum _{k=1}^N\theta _{ik}(H)}, \end{aligned}$$

where by construction $\sum _{j=1}^N{\tilde{\theta }}_{ij}(H)=1$ and $\sum _{i=1}^N\sum _{j=1}^N{\tilde{\theta }}_{ij}(H)=N$.

The total spillover (*SO*) index can be defined as follows:

5 $$\begin{aligned} SO(H)= \frac{\sum _{i=1}^N\sum _{j\ne i}{\tilde{\theta }}_{ij}(H)}{N}. \end{aligned}$$

Similarly, other measures such as “from others” (FO) and “to others” (TO) and “net spillover contribution” (NSO) can be obtained from normalized contribution values:

6 $$\begin{aligned} \text {FO}_{i}= & {} \frac{\sum _{j \ne i} {\tilde{\theta }}_{ji}(H)}{N}, \end{aligned}$$

7 $$\begin{aligned} \text {TO}_{i}= & {} \frac{\sum _{j \ne i} {\tilde{\theta }}_{ij}(H)}{N}, \end{aligned}$$

8 $$\begin{aligned} \text {NSO}_{i}= & {} \text {TO}_{i} - \text {FO}_{i} \end{aligned}$$

where FO ($i \leftarrow j, \, \forall j = 1, 2,..., N, \, j \ne i$) shows to which extent variable *i* receives shocks from all the variables in the system, TO ($i \rightarrow j, \, \forall j = 1, 2,..., N, \, j \ne i$) represents the shock transmission of variable *i* to the whole system, and NSO the difference between TO and FO, representing the net spillover contribution of variable *i* to the system.

Finally, the net pairwise directional connectedness ($\text {C}_{ij}(H)$) is defined as the difference between shocks transmitted from risk category attention/macroeconomic variable *i* to *j* and shocks transmitted from *j* to *i*:

9 $$\begin{aligned} \text {C}_{ij}(H) = \left[ {\tilde{\theta }}_{ij}(H) - {\tilde{\theta }}_{ji}(H) \right] , \, i \ne j. \end{aligned}$$

## Results and discussion

In what follows, we first present and discuss results obtained from a dynamic analysis (Sect. 5.1.1) and from a static analysis (Sect. 5.1.2) on (i) the interaction among global risks (ii) the interaction among global risks and macroeconomic variables. Via standard cross-sectional analyses, we then check whether rising public awareness on most concerning global risks is priced in the cross section of stock returns (Sect. 5.2). Finally, we compare our GRAI with two very popular indicators of uncertainty, i.e., the geopolitical risk index (GPR) of Caldara and Iacoviello (2021) and the economic policy uncertainty index (EPU) of Baker et al. (2016) (Sect. 5.3).

### Global risks awareness, connectedness, and the macroeconomy

In the next section, we present our GRAI and the related net spillover contributions. Forecast error variance decompositions (at 6-months horizon) are retrieved from a VAR(1) estimated using a rolling window of 48 months. The analysis is conducted by relying on (i) only public concerns on the five risk categories (i.e., economic, environmental, geopolitical, societal, and technological) and (ii) public concerns on the five risk categories and macroeconomic variables.

#### Dynamic analysis

**Fig. 2:**
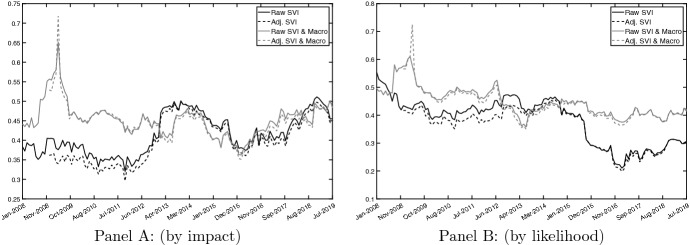
Global Risks Awareness Index (GRAI). *Notes*: Panel **A**: Global risks by impact. Panel **B**: Global risks by likelihood. This figure reports the evolution of the Global Risks Awareness Index (GRAI), captured by the total spillover index computed as described in Eq. (5). Our total spillover metric is based on a VAR(1) model and a forecast horizon of 6 months, computed using a rolling window of 48 months. The GRAI is computed by using (*i*) raw SVIs (solid black line), (*ii*) adj SVIs (dotted black line), (*iii*) raw SVIs & Macrovariables (solid gray line) and (*iv*) adj SVIs & Macrovariables. Raw := Google Trends SVI, Adj := SVI adjusted as in Da et al. (2011). Data are monthly and run from January 2004 to August 2019

Our novel measure of global risks awareness—defined as the total interdependence in the dynamic system of most concerning global risks—is shown in Fig. 2. GRAI based on risks by impact (by likelihood) is shown in Panel A (B). The dynamics shown in Fig. 2 indicate that the population’s concerns for the different categories of risk tend to be highly connected over time. More precisely, we find that, on average, around 50% of the forecast error variance at the 6-month horizon originates from spillovers among the different risk categories. Put it differently, a rising concern in one risk category tends to make people more concerned on other risk types. The inclusion of macroeconomic variables into the system does not alter our main findings, suggesting the presence of a strong interdependence between the different categories of risk and macroeconomic variables. Even if the contribution of spillovers of shocks across global risks to the total forecast error variance is – on average—equal to 50%, its dynamics are strongly time-varying and exhibit some interesting boom and bust periods as well as some peaks. Differences in the dynamics of our GRAI emerge also when (i) risks based on likelihood occurrence instead of risks based on impact are considered (ii) macroeconomic variables are added to the system of initial random variables.

When only concerns on global risks based on impact are considered (Panel A, solid and dashed black lines), the total global risks spillover mostly fluctuates between 35% and 40% over the first five years. It then climbs to 50% in the first half of 2013. It remains stable between 45% and 50% for a couple of years, drops to a value of 40% at the end of 2015, and rises back to 50% in the summer of 2018. Differently, our GRAI fluctuates around 45% over the first five years when macroeconomic variables are accounted for (Panel A, solid and dashed gray lines). Importantly, a rapid increase and a related jump to a value of 70% are observed in the aftermath of the Lehman Brothers collapse, indicating the presence of spillovers between macroeconomic fundamentals and risks perceptions. From 2012 onward, our two measures of interdependence exhibit very similar dynamics (Panel A, black vs. gray lines). Different dynamics in the GRAI are observed when we make use of global risks based on likelihood occurrence. Over the period 2008-2014, our GRAI fluctuates between 40% and 50% and starts declining from the summer of 2014. The total spillover index reaches a minimum value of 20% at the end of 2016 (Panel B, solid and dashed black lines). The inclusion of macroeconomic variables alters, although not significantly, the GRAI’s dynamics. In particular, we still observe a sizable increase in the degree of interdependence induced by macroeconomic fundamentals in the fall of 2018. For the rest of the sample, the GRAI fluctuates between 40% and 50% and does not follow a declining path (Panel B, solid and dashed gray lines), suggesting that business cycle information are positive contributors to total connectedness (i.e., shocks in the fundamentals get transmitted to concerns on global risks).^9^

**Fig. 3:**
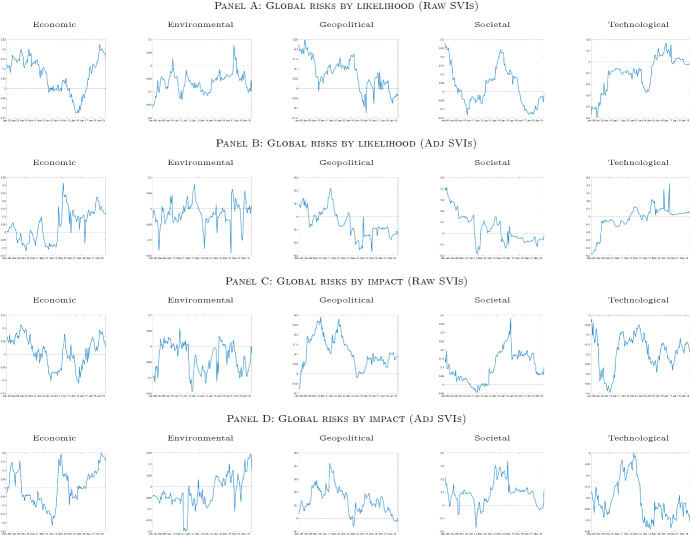
Net Spillover contributions (NSO). *Notes*: The figure reports the net spillover contribution (*NSO*) index based on a VAR(1) model and a predictive horizon for the underlying generalized variance decomposition of 6 months, computed using a rolling window of 48 months. The *NSO* are computed on (*i*) Raw SVIs based on likelihood (Panel **A**), (*ii*) Adj. SVIs based on likelihood (Panel **B**), (*iii*) Raw SVIs based on impact (Panel **C**) and (*iv*) Adj. SVIs based on impact (Panel **D**). Raw := Google Trends SVI, Adj := SVI adjusted as in Da et al. (2011). Data are monthly and run from January 2004 to August 2019

To shed further light on how global risks interact among them as well as with macroeconomic fundamentals, we examine also the dynamics of the net spillover contributions. By doing so, we check whether a variable in the system is a shock transmitter or a shock receiver. The net spillover contributions computed for the system with no macroeconomic variables are shown in Fig. 3. Across the different specifications of our system of variables, rising concerns on economic-, geopolitical-, and societal-related risks are found to be—on average—net transmitters of shocks to other risks. For instance, the public concern of economic-related risk contributes positively to global risks awareness during the period 2008–2011 (see Fig. 3, Panel A, C, and D). An exception is observed when normalized Google searches and global risks by likelihood are employed. In this case, the public concern of economic-related risk is a net transmitter only for the year 2008 (Fig. 3, Panel B). From 2017 onward, across all GRAI specifications, the economic-related risk becomes again a net contributor. Public concern on geopolitical-related risks is also found to be—on average—a net positive contributor to the system during the first part of the sample (i.e., 2008-2014). When referring to normalized Google searches and global risks by likelihood, the contribution of public concern on geopolitical-related risks over the same period is more volatile (Panel B). For instance, in the aftermath of the financial crisis, the geopolitical risk becomes a net receiver. The contribution to the system of public concern of societal-related risks is on—average—positive. The strongest positive contribution is observed during the periods 2008–2009 and 2012–2015.

Differently, public concerns on environmental and technological-related risks are found to be—on average—net shock receivers. In particular, concerns on other risk categories seem to contribute to increasing the concern on environmental-related risks around 2014, i.e., when the contribution of public concern on economic- and societal-related risks is positive. The estimated concern on technological-related risks is constantly a net receiver when the risks by impact are considered (Fig. 3, Panels C and D). Importantly, following a rise in the positive contribution of concern on geopolitical-related risks the negative contribution of technological risk increases in magnitude, indicating that when the general population is more concerned with economic- and geopolitical-related risks, the effect is spread to technological risk. When risks are classified by their likelihood, public concern on technological-related risks is instead a shock receiver until 2013–2014 and starts to become a net positive contributor from 2015 onward (Fig. 3, Panels A and B).

**Fig. 4:**
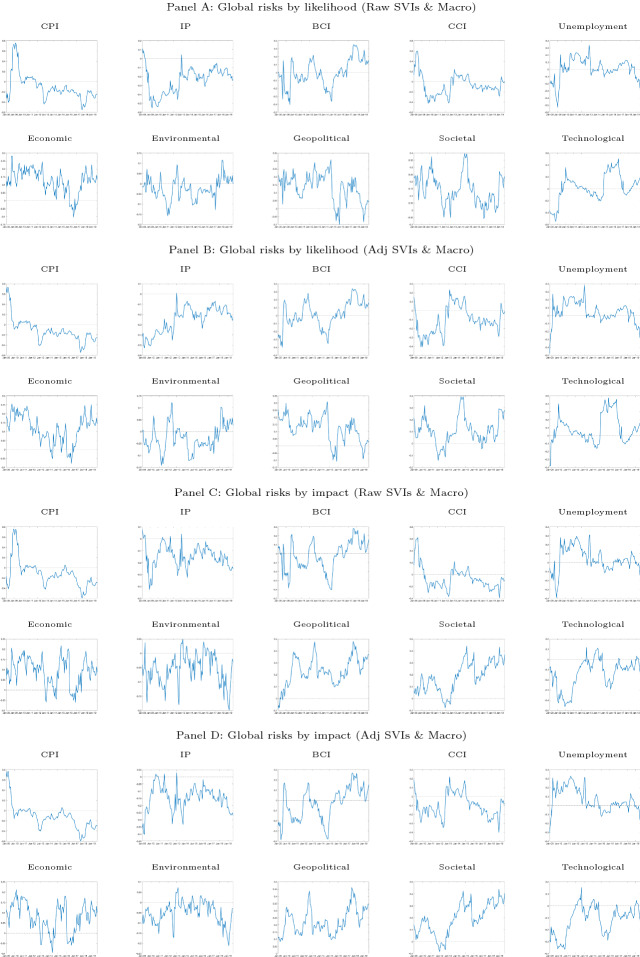
Net Spillover contributions (NSO). *Notes*: The figure reports the net spillover contribution (*NSO*) index based on a VAR(1) model and a predictive horizon for the underlying generalized variance decomposition of 6 months, computed using a rolling window of 48 months. The *NSO* are computed on (*i*) Raw SVIs based on likelihood & Macrovariables (Panel **A**), (*ii*) Adj. SVIs based on likelihood & Macrovariables (Panel **B**), (*iii*) Raw SVIs based on impact & Macrovariables (Panel **C**) and (*iv*) Adj. SVIs based on impact & Macrovariables (Panel **D**). Raw := Google Trends SVI, Adj := SVI adjusted as in Da et al. (2011). Data are monthly and run from January 2004 to August 2019

To better understand how attention to different global risks interacts with the macroeconomy, we also estimate the net spillover contributions for the system composed by both the indicators of public concerns on the five risk categories (i.e., SVI) and the selected macroeconomic variables. These are plotted in Fig. 4 and confirm the dynamics of the net spillover contributions observed in the system composed only by global risks (Fig. 3). In particular, public concerns on economic-, geopolitical-, and societal-related risks are still found to be—on average—shock transmitters.

Let us turn our attention now to the role of macroeconomic fundamentals in the system. Industrial production is found to be a shock receiver over the full period (Fig. 4, Panels A–D). Its net contribution is constantly negative and larger when the positive net contribution of public concern on economic-related risks is larger. One can observe instead the CPI has a mild net contribution to the system. Only from 2015 onward, the CPI starts to have a sizable net negative contribution becoming thus a net receiver.

The overall contribution of business confidence to the system is instead less clear. During and just after the great financial crisis the net contribution fluctuates around zero, indicating that during this period business confidence acts both as shock transmitter and receiver. In other words, attention to most concerning global risks seems to influence business confidence in some periods, while in other periods, business confidence is responsible for rising population’s awareness of global risks. The contribution of BCI to the system is however positive in the last years (i.e., 2017–2019). With respect to the consumer confidence index, its net contribution dynamics are not far from that one of the CPI, at least for some periods. In particular, its net contribution is positive around 2008–2009 and becomes negative thereafter. In other words, for the most part of the analyzed period consumer confidence seems to be affected by the awareness of global risks and other macroeconomic variables. Finally, unemployment is the only macroeconomic indicator that appears to be—on average—a shock transmitter, especially in the first half of our sample. The aftermath of the 2008 collapse, together with the sovereign debt crisis increased unemployment, which in turn generated spillover effects toward other global risks.

Taken together, our evidence suggests that concerns on economic-, geopolitical-, and societal-related risks tend to contribute positively to global risks awareness as well as to macroeconomic fundamentals. In this respect, our results are in line with the recent literature showing that economic-policy-related uncertainty shocks and geopolitical risks represent significant drivers of the macroeconomy, weakening the business cycle and lengthening recovery times after recessions (see, among others, Baker et al. 2016; Caldara and Iacoviello 2021; Ghirelli et al. 2019; Huang and Luk 2020; Donadelli 2015; Shields and Tran 2019).

#### Static analysis

We provide hereafter a static analysis of the directional spillovers among global risks and among global risks and macroeconomic variables in three peculiar periods, i.e., (*i*) the great financial crisis (2007–2009), (*ii*) the sovereign debt crisis and (*iii*) the very last years of our sample. For sake of brevity, we report only the flow of shocks computed using normalized web searches on most concerning global risks (i.e., adj SVIs).

**Fig. 5:**
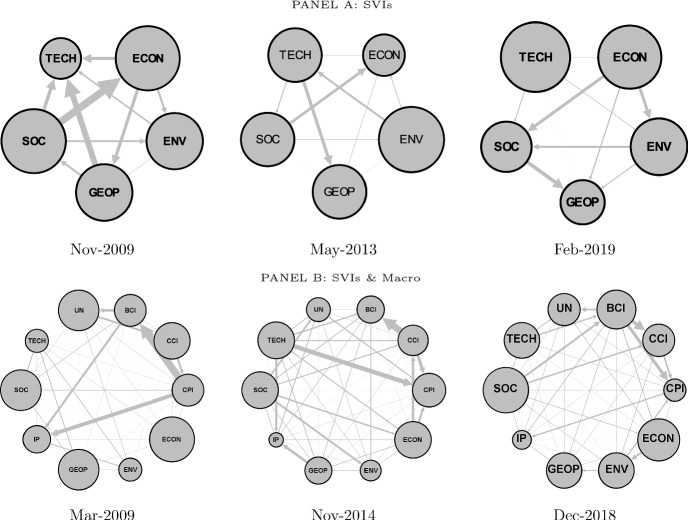
Global Risks Awareness index (GRAI): Global Risks by Likelihood. *Notes*: The figure reports the network graph of the net pairwise directional connectedness based on a VAR(1) and a predictive horizon for the underlying generalized variance decomposition of 6 months, computed using a rolling window of 48 months. The size of the node indicates out-degrees, and the size of the edges indicates the magnitude of the net contribution. Data are monthly and run from January 2004 to August 2019

**Fig. 6:**
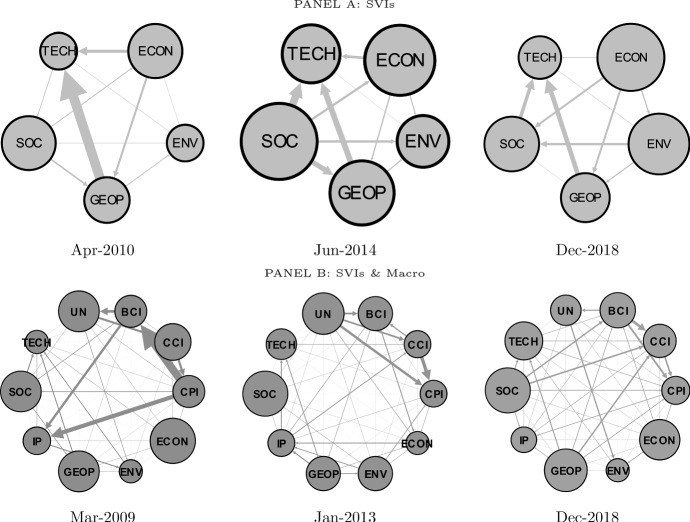
Global Risks Awareness index (GRAI): Global Risks by impact. *Notes*: The figure reports the network graph of the net pairwise directional connectedness based on a VAR(1) model and a predictive horizon for the underlying generalized variance decomposition of 6 months, computed using a rolling window of 48 months. The size of the node indicates out-degrees and the size of the edges indicates the magnitude of the net contribution. Data are monthly and run from January 2004 to August 2019

In the spirit of Diebold and Yılmaz (2009, 2012), for each period, we select the relative peak of the total spillover index (i.e., GRAI). Results for GRAI based on risks by likelihood occurrence (impact) are shown in Fig. 5 (Fig. 6). The size of the nodes is proportional to the out-degree effect (TO) and the size and direction of the edges indicate the magnitude and direction of the net directional connectedness.

Focusing on the global risks classified by likelihood occurrence, we identify November 2009 as the peak for the great financial crisis period (Fig. 5, Panel A). The indicators of attention to economic- and societal-related risk (i.e., SVIs) are found to have the highest TO. One can also observe that concern on societal-related risk heavily influences concern on economic-related risk, whose shock, in turn, is transferred to the remaining three categories of risk. The public concern on geopolitical-related risk is also shown to have a high directional connectedness to public concern on technological-related risk. Differently, in May 2013, a positive net directional connectedness from technological-related risk to the geopolitical-related risk and from societal-related risk to economic-related risk is observed. Despite the underlying economic-policy-related uncertainty induced by the financial crisis and the beginning of the sovereign debt crisis, the public concern on economic-related risk does not represent the one with the strongest out-degree effect nor to have positive net directional connectedness to most of the remaining risk categories. In the most recent period (i.e., February 2019), the public concerns on technological- and economic-related risk are found to have the highest TO. Importantly, concern on economic-related risk is a net transmitter to concerns on environmental- and societal-related risk. The latter then is found to be a net transmitter to concern on geopolitical-related risk.

When global risks classified by impact are considered, we find the public concern on economic-, geopolitical- and societal-related risk to have a higher TO level than concern on environmental- and technological-related risk. In particular, the indicators of public concern on economic-, geopolitical- and societal-related risk are net shock transmitters to the indicators of public concern on environmental- and technological-related risk. An exception is December 2018, during which one can observe that the indicator of public concern on environmental-related risk is a net shock transmitter to public concern on societal-related risk and, to a lower extent, to public concern on geopolitical-related risk. In addition, during this month public concern on environmental-related risk has a higher TO level than concerns on societal- and geopolitical-related risk.

Once macrovariables are taken into account, the prominent role played by the indicators of public concern on economic-, geopolitical- and societal-related risk is confirmed (see Figs. 5 and 6, Panels B). For instance, in November 2014, the levels of public concern on economic- and societal-related risk have a relatively high TO. Interestingly, the indicators of public concern on economic-, environmental- and societal-related risk transmit shocks to industrial production. These in turn spill over to consumer (CCI) and business (BCI) confidence. It is also possible to observe net directional connectedness going from the indicators of concern on the different risk categories to macroeconomic variables (e.g., from the indicator of concern on technological-related risk—with risks classified by likelihood occurrence—to price level in November 2014). Moreover, but not surprisingly, there is high transmission of shocks among macroeconomic variables.

Focusing on the interaction among indicators of public concern on the different types of risk and macrovariables in the last period, one can still observe that public concerns on economic- and societal-related risks represent the main shock transmitters, both having implications for business confidence. The latter transfers then those shocks to consumer confidence and unemployment. Also, attention toward economic-related risk positively contributes to environmental risk attention. Finally, industrial production is the main shock receiver, being influenced by both economic fundamentals (CPI, BCI, and unemployment) and public concern on societal- and geopolitical-related risks.

### Global risks awareness and the cross section of returns

In the spirit of recent empirical studies that examine the asset pricing implications of rising economic-policy-related uncertainty, we test here whether shocks to the degree of connectedness among concerns to different global risks (i.e., GRAI innovations) are priced in the cross section of international returns. To do so, we employ the usual two-step regression approach.

Thus, we first estimate the following regression equation for each asset *i*:

10 $$\begin{aligned} R^{ex}_{i,t} =\alpha _{i}+\beta _{i, \mathrm{MKT}}\mathrm{MKT}_{t}+\beta _{i, \mathrm{SMB}}\mathrm{SMB}_{t}+\beta _{i, \mathrm{HML}}\mathrm{HML}_{t}+\beta _{i,\mathrm{GRAI}}\mathrm{GRAI}_{t}+\epsilon _{i,t} \end{aligned}$$

where $R^{ex}_{i,t}$ is the excess return of stock *i* at time *t*, $\mathrm{MKT}_t$, $\mathrm{SMB}_t$ and $\mathrm{HML}_t$ denote the Fama & French developed factors at time *t*, and the factor $GRAI_t$ represents the interconnectedness of SVIs, or of SVIs and macrovariables, at time *t*, as measured by the previously estimated total spillover index. Finally, $\epsilon _{i,t}$ is the idiosyncratic error term.

**Table 4:** Risk premium of GRAI shocks

	Panel A: GRAI based on Likelihood				Panel B: GRAI based on Impact			
	SVIs		SVIs & MACRO		SVIs		SVIs & MACRO	
	Raw	Adj	Raw	Adj	Raw	Adj	Raw	Adj
Size & BtM	$-$ 2.168***	$-$ 4.623***	0.561**	1.081***	1.158***	3.826***	3.438***	1.910***
	[$-$ 5.305]	[$-$ 6.826]	[2.263]	[4.698]	[3.381]	[10.807]	[10.696]	[7.399]
Size & OP	0.694*	4.806***	0.226	$-$ 0.213	2.936***	6.159***	$-$ 3.745***	$-$ 1.560***
	[1.748]	[7.431]	[0.737]	[$-$0.713]	[7.029]	[10.804]	[$-$ 10.966]	[$-$ 4.594]
Size & INV	0.166	$-$ 0.041	0.336	0.797***	1.051***	3.310***	1.590***	2.296***
	[0.424]	[$-$ 0.064]	[1.346]	[3.321]	[3.225]	[9.315]	[6.114]	[8.767]
Size & Mom	$-$ 0.889**	2.103***	3.447***	1.709***	5.748***	6.844***	2.470***	$-$ 0.529*
	[$-$ 2.122]	[2.938]	[12.174]	[5.673]	[14.292]	[15.110]	[6.620]	[$-$ 1.692]

This table reports the estimated GRAI risk premium from Fama-MacBeth cross-sectional regressions. The sample is based on monthly data from January 2008 to August 2019, and the test assets are 25 developed market portfolios formed on size and Book-to-Market, size and operating profitability, size and investments, and size and momentum (Source: Kenneth R. French Data Library). We consider a four-factor model where the Fama & French factors (market return, SMB, and HML) are used as controls. Raw := Google Trends SVI, Adj := SVI adjusted as in Da et al. (2011). The t statistics in parentheses for the risk premium are adjusted for Shanken correction following Shanken (1992), and for autocorrelation and heteroskedasticity following Newey and West (1987). ***, ** and * denote significance at the 1%, 5% and 10% levels, respectively

In the second step, we compute *T* cross-sectional regressions of returns at each point in time *t* on the previously estimated coefficients of Eq. 10, i.e.,

11 $$\begin{aligned} R^{ex}_{i,t}= \gamma _{i,1}{\hat{\beta }}_{i,\mathrm{MKT}}+\gamma _{i,2}{\hat{\beta }}_{i, \mathrm{SMB}}+\gamma _{i,3}{\hat{\beta }}_{i, \mathrm{HML}}+\gamma _{i,4}{\hat{\beta }}_{i,\mathrm{GRAI}}+\epsilon _{i,t} \end{aligned}$$

In line with the literature studying the price of risk factors (Brogaard and Detzel 2015; Bali and Zhou 2016; Bali et al. 2017; Lee et al. 2021, among others), we correct second stage standard errors for autocorrelation and heteroskedasticity, following Newey and West (1987). Additionally, we follow Cochrane (2009) suggestion and implement the Shanken (1992) correction to account for the sampling error in the $\hat{\beta _i}$. As international portfolios, we use the 25 developed market portfolios formed on size and Book-to-Market, size and operating profitability, size and investments, and size and momentum from Fama&French^10^, as well as the three factors (market return, SMB, and HML) that are used as controls.

Estimated risk premia are reported in Table 4. Entries in Table 4 indicate the presence of a positive and statistically significant risk premium associated with GRAI innovations. Similar results are obtained also when the GRAI is computed by adding macroeconomic variables into the system of random variables. Risk premia, however, tend to be higher in magnitude when only SVIs are considered. In this respect, macroeconomic variables partially offset the risk premium. Our novel evidence is broadly consistent with existing studies finding that rising uncertainty commands a positive risk premium (see, among others, Bekiros et al. 2016; Li 2017).

### Global risks awareness vs. geopolitical risk and policy uncertainty

For the sake of robustness and completeness, we compare our novel indicator measuring the degree of integration among concerns on major global risks (GRAI) with two recently developed and widely used measures of global uncertainty, i.e., (i) the Global Economic Policy Uncertainty (GEPU) index of Baker et al. (2016) and the Geopolitical Risk Index (GPR) of Caldara and Iacoviello (2021). Although both economic- and geopolitical-related risks are embedded in our system, some differences between our GRAI and the GEPU and GPR exist. First and most importantly, our GRAI accounts for more than one category of risk at the same time, whereas the GEPU and GPR rely on one single risk category (economic-policy- or geopolitical-related risk). Second, through the variance decomposition methodology of Diebold and Yılmaz (2009, 2012), our measure of risk actually captures the interconnectedness of concerns among different risks and among different risks and macroeconomic fundamentals, and not just attention on a single specific topic.

**Fig. 7:**
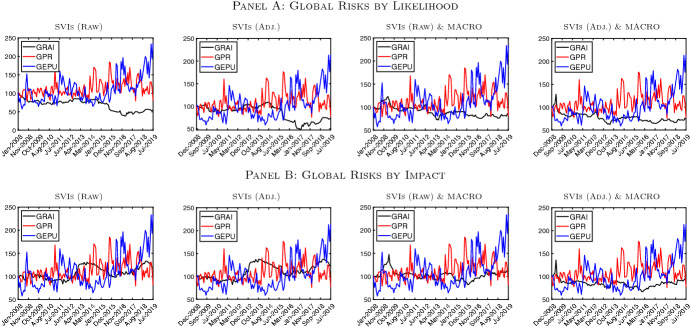
GRAI vs. GPR and GEPU. *Notes*: This figure depicts the evolution of the GRAI [black line], the GEPU of Baker et al. (2016) [blue line] and the GPR of Caldara and Iacoviello (2021) [red line]. In Panel A (B) the GRAI is computed by relying on risks classified by likelihood occurrence (by impact). Raw := Google Trends SVI, Adj. := SVI adjusted as in Da et al. (2011). Data are monthly and run from January 2008 to August 2019

Table 5 shows the correlation between our different versions of GRAI and the GEPU and GPR. Broadly, entries in Table 5 provide evidence of a weak co-movement between the two news-based measures of economic policy-related uncertainty and our indicator of interaction among most concerning global risks. The correlations are all close to zero (or negative) and range from -0.229 (GRAI computed relying on raw SVIs and risks by likelihood occurrence with GPR) to 0.115 (GRAI computed relying on raw SVIs plus macroeconomic aggregates and risks by likelihood occurrence with GPR). We compare our GRAIs’ dynamics with those of the GEPU and GRAI in Fig. 7. The joint dynamics plotted in Fig. 7 confirm the presence of a weak comovement between the two news-based measures of uncertainty and our GRAIs. In particular, one can notice that only in some short periods the pattern of our GRAI follows that of the GEPU or GPR.^11^

**Table 5:** Global risks awareness vs. geopolitical risk and global EPU

Name	Authors	Methodology	Correlation							
			Risks by Likelihood				Risks by Impact			
			SVIs		SVIs & Macro		SVIs		SVIs & Macro	
			Raw	Adj.	Raw	Adj.	Raw	Adj.	Raw	Adj.
GEPU	Baker et al. (2016)	News-based	$-$0.007	$-$0.117	0.115	$-$0.025	0.030	$-$0.033	0.111	0.022
GPR	Caldara and Iacoviello (2021)	News-based	$-$0.229***	$-$0.210**	0.053	0.064	$-$0.014	0.002	0.101	0.103

This table reports estimated correlation coefficients between our GRAIs and the GEPU and GPR. All data are monthly and run from January 2008 to August 2019. ***, ** and * denote significance at the 1%, 5% and 10% levels, respectively

**Table 6:** Risk premia of GEPU and GPR shocks

	Size & BtM	Size & OP	Size & INV	Size & Mom
*Panel* A:				
GPR	$-$ 7.468***	27.862***	7.287***	14.428***
	[$-$ 4.647]	[13.285]	[4.472]	[8.612]
*Panel* B:				
GEPU	9.613***	$-$ 0.029	$-$ 9.212***	12.734***
	[5.292]	[$-$ 0.015]	[$-$ 5.127]	[7.587]

This table reports estimated GPR (Panel A) and GEPU (Panel B) risk premia from Fama-MacBeth cross-sectional regressions. The sample is based on monthly data from January 2008 to August 2019, and the test assets are 25 developed market portfolios formed on size and Book-to-Market, size and operating profitability, size and investments, and size and momentum (Source: Kenneth R. French Data Library). We consider a four-factor model where the Fama & French factors (market return, SMB, and HML) are used as controls. The t statistics in parentheses for the risk premium are adjusted for Shanken correction following Shanken (1992), and for autocorrelation and heteroskedasticity following Newey and West (1987). ***, **, and * denote significance at the 1%, 5%, and 10% levels, respectively

For the sake of robustness, we also estimate the risk premia of GEPU and GPR shocks. Results from the second stage regression are reported in Table 6. For developed portfolios sorted by size and moments both GPR and GEPU shocks are found to carry a positive risk premium. GPR shocks command a positive risk premium also across portfolios sorted on (i) size and operating profitability and (ii) size and investments. Taken together, results from cross-sectional asset pricing tests indicate that both rising connectedness among concerns on major global risks (i.e., economic, environmental, geopolitical, societal, and technological) and mounting media coverage on economic-policy- and geopolitical-related issues are priced in the cross section of international returns and carry—on average—positive risk premia.

## Concluding remarks

In this paper, we analyze the relationship between the macroeconomy and public awareness of five major global risk categories identified by the World Economic Forum (i.e., economic, environmental, geopolitical, societal, and technological). Following the FEVD approach (Diebold and Yılmaz, 2009, 2012), we propose a novel index of Global Risks Awareness (GRAI) to measure spillover effects arising among public concerns on the five different categories of risk. We capture public concern on each risk category by using Google search volume indexes (SVIs) on specific keywords/topics.

Overall, we find a significant degree of spillover among the five SVIs that persists after the inclusion of macroeconomic variables such as the unemployment rate, the industrial production index, and the consumer price index. Interestingly, the interconnectedness among SVIs and the macroeconomy is decreasing over time, suggesting that perceptions of the five risks are progressively deviating from the underlying macroeconomic fundamentals. On average, we find that the five SVIs are net positive contributors to the system, while macroeconomic variables are net receivers. Both dynamic and static analyses provide evidence that shocks on economic-, geopolitical-, and societal-related risk perceptions have a role in forming economic expectations and exert an influence on the considered macroeconomic variables. Finally, we find that the degree of interconnectedness among major global risks is priced in the cross section of international returns. Using different sorting for the 25 developed markets portfolios of Fama and French, we show that a rise in the level of awareness of major global risks carries positive risk premia. Further investigations at a granular level to improve the identification of specific risk drivers triggered by abrupt changes in geopolitical, societal, and climate scenarios are certainly welcome.

## Figures and Tables

**Fig. 8:**
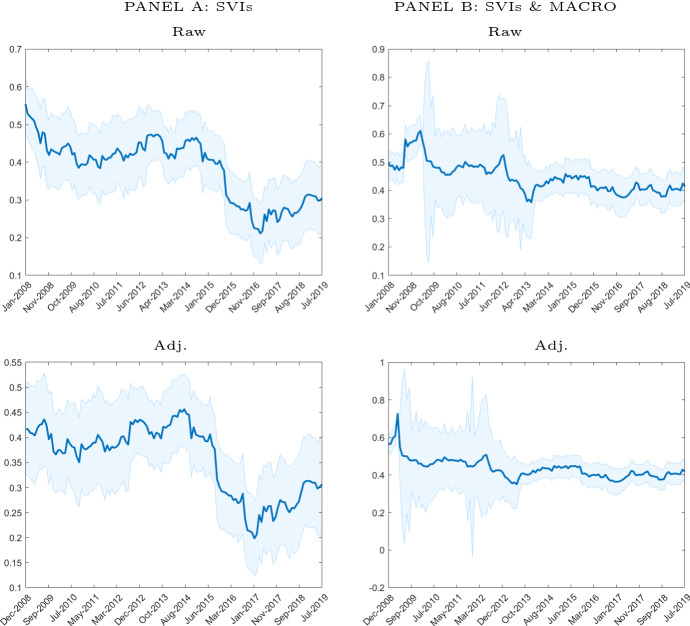
Global Risks Awareness index (GRAIs): Risks by Likelihood. *Notes*: The figure reports GRAI, corresponding to the Spillover (*SO*) index based on a VAR(1) model and a predictive horizon for the underlying generalized variance decomposition of 6 months, computed using a rolling window of 48 months. Panel A shows GRAI computed on SVIs (based on likelihood) only, and Panel B shows GRAI computed on SVIs (based on likelihood) and Macro variables. Raw := Google Trends SVI, Adj := SVI adjusted as in Da et al. (2011). Standard errors are robust to 1000 bootstrap replications, following Bostanci and Yilmaz (2020). The blue shaded area indicates 90% statistical confidence. Data are monthly and run from January 2004 to August 2019

**Fig. 9:**
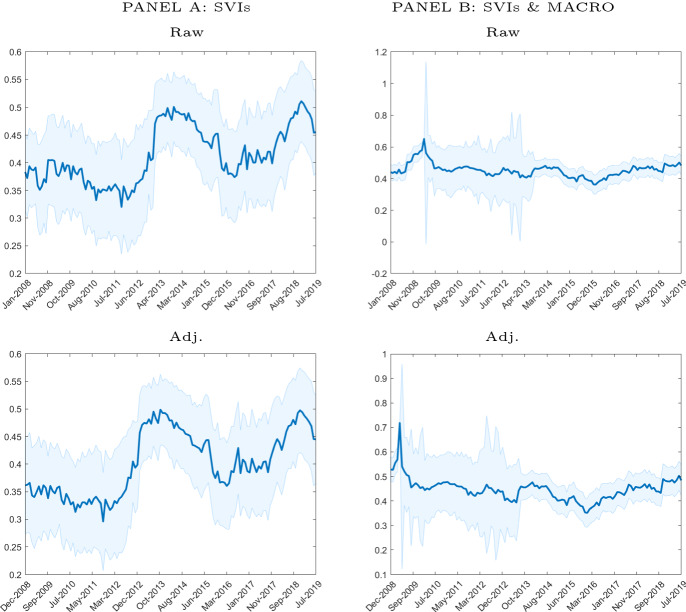
Global Risks Awareness index (GRAIs): Risks by Impact. *Notes*: The figure reports GRAI, corresponding to the spillover (*SO*) index based on a VAR(1) model and a predictive horizon for the underlying generalized variance decomposition of 6 months, computed using a rolling window of 48 months. Panel **A** shows GRAI computed on SVIs (based on impact) only, and Panel **B** shows GRAI computed on SVIs (based on impact) and macrovariables. Raw := Google Trends SVI, Adj := SVI adjusted as in Da et al. (2011). Standard errors are robust to 1000 bootstrap replications, following Bostanci and Yilmaz (2020). The blue shaded area indicates 90% statistical confidence. Data are monthly and run from January 2004 to August 2019

## Data Availability

Data are available upon reasonable request.

## Appendix

**Table 7:** Vertical aggregation: an example

	2010–01	2010–02	2010–03	2010–04	2010–05	2010–06
People’s Republic of China economy	1.0	0.5	1.0	0.7	1.0	0.5
Financial market	1.0	1.0	1.0	1.0	2.0	1.0
Fiscal imbalance	2.0	2.0	2.0	2.0	3.0	2.0
Globalization	8.0	9.0	11.0	11.0	11.7	9.0
Natural gas	18.0	17.0	17.3	17.0	16.0	17.0
Petroleum	36.7	37.7	40.7	47.3	98.7	99.7
Unemployment	67.3	63.0	67.7	62.0	57.7	65.0
**Economic “RAW” SVI (Impact)**	**134.0**	**130.2**	**140.7**	**141.0**	**190.0**	**194.2**

This table reports some examples of vertical aggregation for a small subset of months only. The value of Economic “raw” SVI [last row, bold] is obtained summing, for each period, the search volumes of topics belonging to the risk category ‘Economic’

**Table 8:** Summary statistics

Panel A: “Raw” SVI (impact)							
	Min	1st Qu.	Median	Mean	3rd Qu.	Max	St.dev
Economic	0.384	0.502	0.580	0.592	0.675	1	0.116
Environmental	0.277	0.398	0.533	0.514	0.604	1	0.132
Geopolitical	0.048	0.066	0.074	0.095	0.087	1	0.094
Societal	0.350	0.424	0.481	0.501	0.541	1	0.112
Tecnological	0.055	0.093	0.128	0.202	0.251	1	0.170

Panel B: “Raw” SVI (likelihood)							
	Min	1st Qu.	Median	Mean	3rd Qu.	Max	St.dev
Economic	0.384	0.502	0.580	0.592	0.675	1	0.116
Environmental	0.308	0.412	0.505	0.506	0.566	1	0.108
Geopolitical	0.294	0.391	0.440	0.491	0.564	1	0.142
Societal	0.312	0.401	0.448	0.471	0.504	1	0.107
Tecnological	0.040	0.059	0.074	0.088	0.091	1	0.079

Panel C: “Adjusted” SVI (impact)							
	Min	1st Qu.	Median	Mean	3rd Qu.	Max	St.dev
Economic	0.750	0.919	0.967	0.984	1.035	1.440	0.106
Environmental	0.808	0.966	1.025	1.056	1.117	1.691	0.143
Geopolitical	0.472	0.818	0.959	0.941	1.079	1.263	0.163
Societal	0.718	0.905	0.983	0.997	1.046	1.658	0.150
Tecnological	0.550	0.823	0.942	0.936	1.042	1.462	0.169

Panel D: “Adjusted” SVI (likelihood)							
	Min	1st Qu.	Median	Mean	3rd Qu.	Max	St.dev
Economic	0.750	0.919	0.967	0.984	1.035	1.440	0.106
Environmental	0.747	0.954	1.011	1.059	1.138	2.006	0.173
Geopolitical	0.765	0.896	0.973	0.965	1.028	1.927	0.119
Societal	0.690	0.904	0.989	0.992	1.047	1.699	0.156
Tecnological	0.596	0.882	0.979	1.156	1.144	12.625	1.018

This table reports main summary statistics for ”Raw" SVI by impact (Panel A), ”Raw" SVI by likelihood (Panel B), ”Adj." SVI by impact (Panel C) and ”Adj." SVI by likelihood, for each risk category: (*i*) Economic, (*ii*) Environmental, (*iii*) Geopolitical, (*iv*) Societal and (*v*) Technological. Raw;) Google Trends SVI, Adj. := SVI adjusted as in Da et al. (2011). Data are monthly and run from January 2004 to August 2019

## Footnotes

1 Throughout the paper, the terms awareness and concern will be used interchangeably.

2 In Google trends, the time series related to a specific search term are related to that very specific word (or combination of words) queried. Therefore, translations in other languages or synonymous are not considered. Moreover, the time series are sensitive to the use of the singular or plural (e.g., searching for *paper* or for *papers* produces two different time series). A similar approach relying on Google “topics” has been used by Kupfer and Zorn (2020) to develop a Google search-based EPU index for Eastern Europe.

3 The “related queries” and “related topics” function of Google Trends, available for each topic, are helpful to check whether a given topic is somewhat related to that specific factor.

4 For example, if we want to capture concerns on the global risk “*water supply crises*,” the topic “*water supply*” exists. However, an analysis of the “related queries” and “related topics” in Google Trends reveals that it is unrelated to *crises*. Hence, we drop the topic “*water supply*” and apply the second criterion.

5 The keyword for the topic “People’s Republic of China economy” does not work when Google Trends is set in English. The search volume for the topic “underemployment” is found to be quantitatively irrelevant, and for this reason, excluded.

6 Google actually provides SVIs that are bounded between 0 and 100: SVI assumes value 100 for the observation with the highest search volume, with the remaining values that are proportionally scaled. Thus, retrieving SVIs for more than one search term or topic with the same query, it is possible to compare the distribution and the average search volume of multiple keywords.

7 An example of the employed vertical aggregation is provided in the Appendix (see Table 7).

8 Note that SVIs retrieved from Google Trends have some drawbacks. In particular, Google SVIs for the same search downloaded at different points in time may differ (Bontempi et al. 2021; Da et al. 2011; Donadelli and Gerotto 2019; Shields and Tran 2019). In the spirit of Donadelli and Gerotto (2019), we fix this issue by downloading the same SVI several times across different days. The average is then used as benchmark SVI. By doing so, we make the residual randomness economically negligible.

9 For the sake of robustness, we estimate and add bootstrap confidence bands to our measure of total interdependence among concerns to different risk categories. Bootstrap confidence bands are computed as in Bostanci and Yilmaz (2020). Results from this additional test are reported in Figs. 8 and 9.

10 Data have been downloaded from Kenneth French’s website (https://mba.tuck.dartmouth.edu/pages/faculty/ken.french/data_library.html)

11 Let us stress that our GRAIs are computed in a rolling-window fashion and for this reason exhibit much smoother dynamics than the GPR and GEPU that are computed by counting daily news.

## Declarations

### Code availability

Code is available upon reasonable request.
